# Advances in MS Based Strategies for Probing Ligand-Target Interactions: Focus on Soft Ionization Mass Spectrometric Techniques

**DOI:** 10.3389/fchem.2019.00703

**Published:** 2019-10-23

**Authors:** Guilin Chen, Minxia Fan, Ye Liu, Baoqing Sun, Meixian Liu, Jianlin Wu, Na Li, Mingquan Guo

**Affiliations:** ^1^Key Laboratory of Plant Germplasm Enhancement and Specialty Agriculture, Wuhan Botanical Garden, Chinese Academy of Sciences, Wuhan, China; ^2^Sino-Africa Joint Research Center, Chinese Academy of Sciences, Wuhan, China; ^3^Graduate University of Chinese Academy of Sciences, Beijing, China; ^4^State Key Laboratory of Respiratory Disease, National Clinical Center for Respiratory Diseases, Guangzhou Institute of Respiratory Diseases, First Affiliated Hospital, Guangzhou Medical University, Guangzhou, China; ^5^State Key Laboratory for Quality Research of Chinese Medicines, Macau University of Science and Technology, Taipa, Macau

**Keywords:** ligand-target interactions, mass spectrometry, soft ionization, drug discovery, ESI-MS, MALDI-MS

## Abstract

The non-covalent interactions between small drug molecules and disease-related proteins (ligand-target interactions) mediate various pharmacological processes in the treatment of different diseases. The development of the analytical methods to assess those interactions, including binding sites, binding energies, stoichiometry and association-dissociation constants, could assist in clarifying the mechanisms of action, precise treatment of targeted diseases as well as the targeted drug discovery. For the last decades, mass spectrometry (MS) has been recognized as a powerful tool to study the non-covalent interactions of the ligand-target complexes with the characteristics of high sensitivity, high-resolution, and high-throughput. Soft ionization mass spectrometry, especially the electrospray mass spectrometry (ESI-MS) and matrix assisted laser desorption ionization mass spectrometry (MALDI-MS), could achieve the complete transformation of the target analytes into the gas phase, and subsequent detection of the small drug molecules and disease-related protein complexes, and has exerted great advantages for studying the drug ligands-protein targets interactions, even in case of identifying active components as drug ligands from crude extracts of medicinal plants. Despite of other analytical techniques for this purpose, such as the NMR and X-ray crystallography, this review highlights the principles, research hotspots and recent applications of the soft ionization mass spectrometry and its hyphenated techniques, including hydrogen-deuterium exchange mass spectrometry (HDX-MS), chemical cross-linking mass spectrometry (CX-MS), and ion mobility spectrometry mass spectrometry (IMS-MS), in the study of the non-covalent interactions between small drug molecules and disease-related proteins.

## Introduction

The life process is closely related to numerous inherent biological macromolecules, such as proteins, peptides, and nucleic acids, which are important components of the biological organisms. These macromolecules, on the one hand, could regulate the body‘s signal transduction among cells and maintain the normal substantial and energy metabolism to play vital roles in the life activities. On the other hand, a large proportion of small drug molecules take effects by majorly interacting with these disease-related drug targets in the pharmacological process of treatment (Mulabagal and Calderón, 2010; Chen et al., 2018a). Thereof the non-covalent interactions between small drug molecules and biological macromolecules have always been a hot topic in medicinal chemistry and life sciences worldwide. The non-covalent interactions, including the hydrogen bond, Van der Waals forces, electrostatic interactions and hydrophobic effects, between small drug molecules and biological macromolecules are very weak, which are generally <10 kJ·mol^−1^ and 1–2 magnitudes smaller than the usual covalent bond with the range of action from 0.3 to 0.5 nm (Zhang and Abliz, 2005). However, these weak intermolecular interactions could exert additive and synergistic effects under certain conditions, and further form some kinds of strong forces with directivity and selectivity. To some extent, the studies of the properties of these forces could strongly help to understand the interactions between molecules and their mutual recognition process in the field of life sciences, medicine, and pharmacy (Ramos and Santana-Marques, 2009; Han et al., 2018). Therefore, the detection and characterization of the non-covalent complexes have become a commonly concerned hot issue.

Currently, the determination methods for studying the non-covalent complexes mainly include spectral methods [UV, IR, fluorescence, circular dichroism (CD), etc.], chromatography, hypervelocity centrifugation, NMR, and X-ray crystal diffraction, which have their own advantages and disadvantages (Mulabagal and Calderón, 2010). The spectral methods could provide the information of the structural changes before and after the formation of complexes, while little or no information on the relative molecular weight (*M*_*w*_) and stoichiometric ratio of complexes (Zhang and Abliz, 2005). The X-ray crystal diffraction method can only be used when suitable crystal is obtained (Wilderman et al., 2010); the NMR method requires a large number of samples, and is inappropriate for those samples with the *M*_*w*_ >30 ku (Marion, 2013).

Mass spectrometry (MS) has been characterized by the high sensitivity, rapidity, and specificity (Ma et al., 2016; Zhu et al., 2018). Recently, the development of soft ionization technologies, especially the electrospray ionization (ESI) and matrix assisted laser desorption ionization (MALDI), has extended the analysis range of MS from small molecules to biological macromolecules (Yao et al., 2013). During the assay, MS could provide a large number of stoichiometric and spectral information with small sample consumption (*p*mol - *f* mol), which makes MS show great advantages in studying the non-covalent complexes. For example, due to its soft ionization conditions, soft ionization MS will not be limited by the solubility and *M*_*w*_ in the study of the interactions between small drug molecules and biological macromolecules (Dettmer et al., 2007). Furthermore, the soft ionization MS can be applied directly to obtain the stoichiometric ratios between drugs and biological macromolecules, calculate the binding strength between the ligand-protein complexes, determine the binding site of drugs, and obtain the reaction kinetics and others (Bolbach, 2005; Hofstadler and Sannes-Lowery, 2006). In addition, unlike the NMR or CD techniques that measure the average properties of biological macromolecules, soft ionization MS coupled with hydrogen/deuterium (H/D) exchange techniques could quantitatively describe the protein folding dynamics (Winston and Fitzgerald, 1997; Ramirez-Sarmiento and Komives, 2018). Finally, MS can be easily combined with various chromatographic techniques, which is very suitable for studying the interactions between various small drug molecules and biological macromolecules in complex systems (Zinn et al., 2012; Guo et al., 2017).

Drug targets commonly refer to the biological macromolecules existing in tissues and cells that exhibit specific interactions with drug molecules and enable drugs to exert their expected biological activities, and more than 95% of which are the proteins, including enzymes, receptor proteins, ion channel proteins, regulatory factors, and nuclear receptors (Evans and Relling, 1999; Gao et al., 2008). Therefore, to accurately explain and describe the ligand-target interactions is not only the key scientific problem for the drug development, but also the most challenging frontier scientific issue in chemical biology, especially in chemical genomics (Sato et al., 2007). In this regard, many new methods and technologies for the detailed interpretation of the ligand-target interactions derived from modern analytical techniques have been brought into being, among which MS and its hyphenated technologies, including but not limited to the cross-linking MS (CX-MS) (Ferraro and Cascio, 2018), hydrogen-deuterium exchange MS (HDX-MS) (Ramirez-Sarmiento and Komives, 2018), ion mobility MS (IM-MS) (Goth and Pagel, 2017), and hydrophilic interaction chromatography MS (HILIC-MS) (Jin et al., 2017), are the most widely used technologies for studying the interactions between small drug molecules and biological macromolecules. To this end, this present manuscript summarized and reviewed the applications of the soft ionization MS, especially the ESI-MS and MALDI-MS, in the study of the interactions between small drug molecules and biological macromolecules.

## Soft Ionization Ms Techniques For Probing The Non-Covalent Interactions

**Figure 1:**
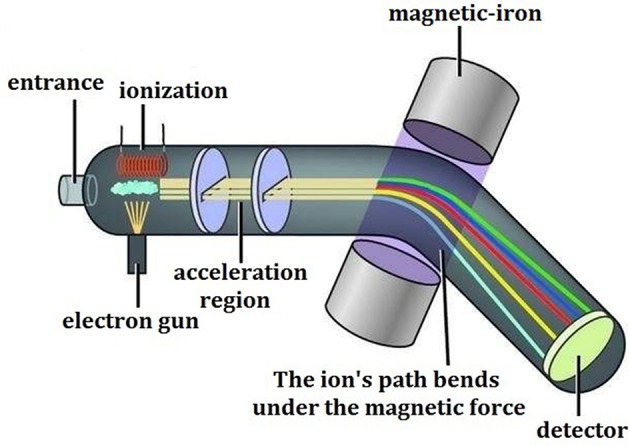
The schematic diagram of MS.

Mass spectrometry, as its name implies, refers to the procedures that after the samples are converted into moving gaseous ions, a variety of charged ions will be separated from each other according to their own specific mass/charge ratio (m/z) and then form their own different motion tracks in a high vacuum mass analyzer with applied electric field or magnetic field, and the final mass spectrogram is generated through data recording and conversion. The corresponding technology and instrument are called as the mass spectrometer (Figure 1), which generally consists of five parts, including the sampling system, ion source, mass analyzer, detector, and data processing system.

In the late 1980s, the soft ionization technologies represented by ESI and MALDI have opened up a new field for the MS, which can be widely applied to analyze the biological macromolecules, synthetic polymers and other thermal unstable, strong polar, and volatile compounds that cannot be detected by the conventional MS. Under certain conditions, non-covalent complexes can be “intactly” transformed from the solution to the gas phase and then directly detected by soft ionization method, which can provide the valuable information of the stoichiometric ratios, the binding types and energies of the ligand-target complexes. In 1991, Ganem and his partners firstly employed the ionspray (similar to ESI) MS to investigate the non-covalent interactions of the complexes formed by the immune protein FKBP and immune drug FK506, and found that the determination conditions strongly affected the ionic strength of the complexes, while the compositions of the complexes were independent of the drug concentration in solution, and the stoichiometric ratio was 1:1 (Ganem et al., 1991). Since then, the ESI-MS and MALDI-MS have been used successfully in the protein and peptide detection and sequencing, DNA sequencing, protein folding, evaluation of the contribution of individual amino acid residues to protein function, *in vitro* drug analysis and new drug research and development (Siuzdak, 1994; Hofstadler and Sannes-Lowery, 2006; Ramos and Santana-Marques, 2009).

## ESI-MS and Its Applications in Ligand-target Interactions

### Characteristics of the ESI-MS

**Figure 2:**
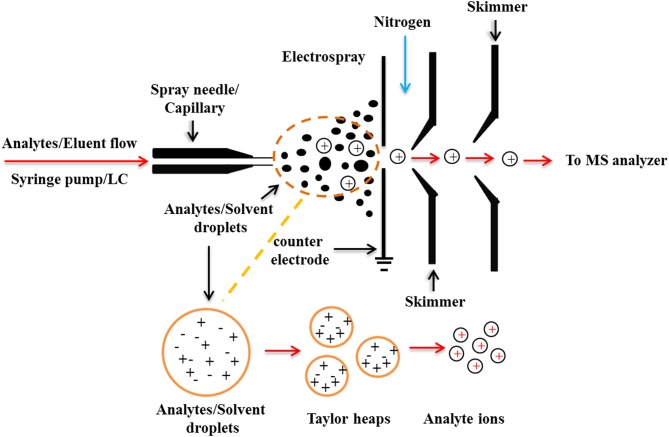
The schematic workflow of typical ESI ionization processes.

The ESI process can be generally divided into three stages: droplet formation, droplet atrophy, and gaseous ion formation (Figure 2). During the ionization process of ESI, the solute molecules are not attacked by other molecules or particles such as atoms, ions, etc., thus maintaining a “complete” molecular ion into the mass spectrometer. Distinguished from the classical EI source, the ESI produces molecular ions with multiple charges, which broadens the mass range of mass spectrometry detection. Based on the soft ionization characteristics of the ESI source, Loo summed up the 4 “S” characteristics of the ESI-MS: Sensitivity, Speed, Specificity, and could directly give the Stoichiomotry (Loo, 1997).

The soft ionization process of ESI-MS also allows the super-molecular complexes bound with weak non-covalent interactions to be completely detected, which could directly predict the chemical binding quantitative relationships of each component of the complexes, thus providing important structural information for the study of super-molecular systems. In addition, the ESI-MS can measure non-covalent complexes from the solution phase into the gas phase, which is very close to the state of the natural solution and could truly reflect the aggregation state of the test molecules in the solution. Therefore, it plays an important role in the study of the non-covalent complexes of the molecular recognition, and is an ideal analytical tool for studying the interactions between small molecules and biological macromolecules. For example, the interactions of the protein-protein, DNA-protein, DNA-drug, and antibody-antigen complexes have been successfully studied using ESI-MS (Liang et al., 2018).

### Applications of ESI-MS in the Interactions of Small Drug Molecules With DNA

DNA is the main carrier of genetic information in living organisms. A great number of drugs interact with DNA by interfering with the regulation and expression of disease-related genes, making DNA the target of many drugs in human body (Brodbelt, 2010; Mirzaei et al., 2013; Chan et al., 2017). Therefore, the study of the interactions between drug molecules and DNA not only helps to understand the mechanisms of action of drugs at the molecular level, but also has important theoretical guiding significance for the screening of drugs *in vitro* and the design of the disease-resistant drugs. Table 1 presents some representative studies and findings in this field.

**Table 1:** Representative studies of ESI-MS technologies for the non-covalent interactions between small drug molecules and DNA.

**Subjects**	**Ligands**	**Type of MS**	**References**
Single/double-stranded DNA	EGCG	CSI-MS	Kuzuhara et al., 2006
Hairpin DNA	Chelerythrine, sanguinarine	ESI-TOF MS	Bai et al., 2008
DNA duplexes/triplexes	Mitoxantrone	ESI-FT-ICR MS	Wan et al., 2008
Double-stranded oligodeoxynucleotides	Distamycin, hoechst 33258, hoechst 33342, berenil	ESI-MS	Wan et al., 2000
Three double-stranded oligodeoxynucleotides	Five bis-β-carbolines alkaloids	ESI-FT-ICR MS	Dong et al., 2007
Quadruplex DNA	Ditercalinium	ESI-TOF MS	Carrasco et al., 2002
EthR6-DNA and EthR4-DNA	Ethionamide	NanoESI-Q-TOF MS	Chan et al., 2017

In this research area, Wan et al. (2000) observed the competitive binding effects of four drugs (distamycin, hoechst 33258, hoechst 33342, and berenil) to one double stranded DNA, and calculated the relative affinity strength of the same pair of stranded DNA according to the abundance ratio of the unbound double stranded DNA and the bound double stranded DNA in ESI-MS. Results suggested that the binding strengths of the four drug molecules to DNA was: hoechst 33342 > hoechst 33258 > distamycin > berenil. In another effort, Carrasco et al. (2002) confirmed the preferential interaction between the antitumor drug ditercalinium and DNA with ESI-MS, and analyzed the ligation reaction in detail by surface plasmon resonance (SPR) spectroscopy, which provided a new idea for the design of drugs that can be designed to be ligated to the human tolemeres. Besides, Dong et al. (2007) studied the interactions between five bis-β-carboline alkaloids and three double-stranded DNA. The results showed that the length of the connecting arm in the drug chemical structures could affect the strength of its interaction with DNA, and too short or too long of the connecting arm would weaken the binding ability of alkaloids to DNA. Furthermore, Bai et al. (2008) used ESI-MS to study the interactions between chelerythrine, sanguinarine, and a series of hairpin DNA. The results showed that the two alkaloids exerted strong binding affinities with the hairpin DNA molecules containing a pyrimidine swelled part; and the interaction between sanguinarine and DNA was stronger than that of the chelerythrine, while the sequence selectivity of the latter was higher than that of the former.

In addition, coldspray ionization (CSI) is a novel ionization method based on an improved ESI ion source (Yamaguchi, 2003). With this improved technique, the CSI-MS has shown promising application prospects in the analysis of the self-assembled nano-scale organometallic complexes structures, DNA complexes structures, weak interactions between organic molecules and nucleosides, and the formation of the molecular clusters (Sakamoto et al., 2000; Sakamoto and Yamaguchi, 2003). For instance, Kuzuhara et al. (2006) used CSI-MS to study the interactions between the (-)-epigallocatechin gallate (EGCG) and the single-stranded DNA, single-stranded RNA and double-stranded DNA with the green tea extracts, and the results showed that the galactyl and catechin groups in EGCG structure contributed to the binding affinities to nucleic acid molecules.

### Applications of ESI-MS in the Interactions of Small Drug Molecules With Proteins

Protein is also one of the main targets for drugs to exert pharmacological effects in human body. On the one hand, drugs can regulate and change the biological functions of receptor proteins by combining with the receptor proteins, such as ion channel and enzymes, which could change the spatial conformation of the receptor proteins or compete with the natural ligand of the receptor proteins, so as to exert their pharmacodynamic effects (Evans and Relling, 1999). On other hand, through the combination with the human serum albumin (HSA), drugs can be delivered to the tissues and organs where they work, which could also effectively control the drug release and prevent the rapid drug metabolism (Iwao et al., 2018; Tzameret et al., 2019). Therefore, the studies on the interactions between small drug molecules and protein targets shown in the following Table 2, not only help to understand the mechanisms of pharmacological action of drugs at the molecular level, but also help to fully evaluate the absorption, distribution, metabolism, and excretion of drugs in the body.

**Table 2:** Representative studies of ESI-MS technologies for the non-covalent interactions between small drug molecules and proteins.

**Subjects**	**Ligands**	**Type of MS**	**References**
hGHbp	Six nonpolar ligands	ESI-Q-TOF MS	Tjernberg et al., 2004
P-glycoprotein	Cyclosporin A and charged/zwitterionic lipids	IM-MS	Marcoux et al., 2013
Chorismate mutase	Adamantyl-1-phosphonate	ESI-TOF MS	Wendt et al., 2003
Ribonuclease A	Cytidine 2'-monophosphate, cytidine triphosphate	NanoESI-Q-TOF MS	Zhang et al., 2003
Norovirus P domain	41 HBGA oligosaccharides	FT ICR-NanoESI-MS	Han et al., 2013
α_1_-Acid glycoprotein	Propranolol, pindolol, oxprenolol, alprenolol, carbamazepine, warfarin	CE/FA-ESI-MS	Vuignier et al., 2012
BSA	Warfarin, salicylic acid, diclofenac	CE/FA-ESI-MS	
Carbonic anhydrase II (CA II)	Furosemide, acetazolamide, 4-CBSA, DNSA, sulfanilamide, and Sulpiride	MIK-MS	Obi et al., 2018
bCA II	Eighty-five methanolic plant extracts	Online SEC-ESI-FTICR-MS	Vu et al., 2008
HSA	Tanshinon IIA, warfarin	ESI-TOF MS	Liu et al., 2008
β-Lactoglobulin	Morin, quercetin, myricetin	HPLC-ESI-Q-TOF MS	Xu et al., 2018
COX-2	Flavonoids in lotus plumule	UF-LC/ESI-MS	Chen et al., 2019
COX-2, Top I	*R. davurica* extracts	UF-LC/ESI-MS	Chen et al., 2018b

Chen et al. (2004, 2008) phosphorylated the flavonoids of myricet, myricetin, and 7-hydroxy flavone, and then compared the interactions between these flavonoids and their related phosphorylated derivatives with proteins by ESI-MS. The results revealed that the binding strength of acylated flavonoids with proteins was stronger than that of flavonoids, and the *in vitro* assays also proved that the former displayed stronger inhibitory activity on tumor cells than the latter. Liu et al. (2008) investigated the effects of tanshinone IIA on the interaction between the anticoagulant drug warfarin and HAS by using ESI-MS. It was found that tanshinone IIA could competitively bind to the HSA with warfarin, which not only resulted in the increase of the free warfarin in the blood, but also accelerated the metabolism of warfarin, and thus this phenomenon was very helpful to elucidate how the tanshinone IIA enhanced the pharmacological effects of warfarin in the body at the molecular level.

β-Lactoglobulin (β-G) exists extensively in the milk of most mammals and is the main component of whey protein. It has good biodegradability and adaptability and can be used as an excellent carrier (Mensi et al., 2013). In this regard, Xu et al. (2018) studied the interactions between flavonoids (morin, quercetin and myricetin) and β-lactoglobulin by high performance liquid chromatography-electrospray-quadrupole-time-of-flight high-resolution mass spectrometry (HPLC-ESI-Q-TOF MS). The binding constants of the β-lactoglobulin interacting with three flavonoids at different volume ratios were determined. The results showed that morin, quercetin and myricetin could form stable complexes with β-lactoglobulin at different volume ratios; the number of the flavonoid molecules bound with a single β-lactoglobulin increased with the decrease of the volume ratio (the proportion of small flavonoid molecules increased); the complexes exhibited strong binding abilities when the binding constant order of magnitude was 10^3^-10^5^, and the binding ability of the three flavonoid molecules and β-lactoglobulin was of the order: morin> quercetin> myricetin.

Affinity ultrafiltration mass spectrometry (UF-LC/MS) is a high-throughput (HTS) technique for the fast screening and identification of the active components from the complex mixtures, such as the medicinal plant extracts and compounds libraries. Targeted to the key proteins in the process of disease occurrence and development, this technique could effectively fish out the active compounds from the unbound inactive components in the complex mixtures based on the biological affinities between the small molecule compounds and the targeted proteins in the body, and thus has the characteristics of high sensitivity and rapidity (Li et al., 2015; Qin et al., 2015; Chen et al., 2018b).

**Figure 3:**
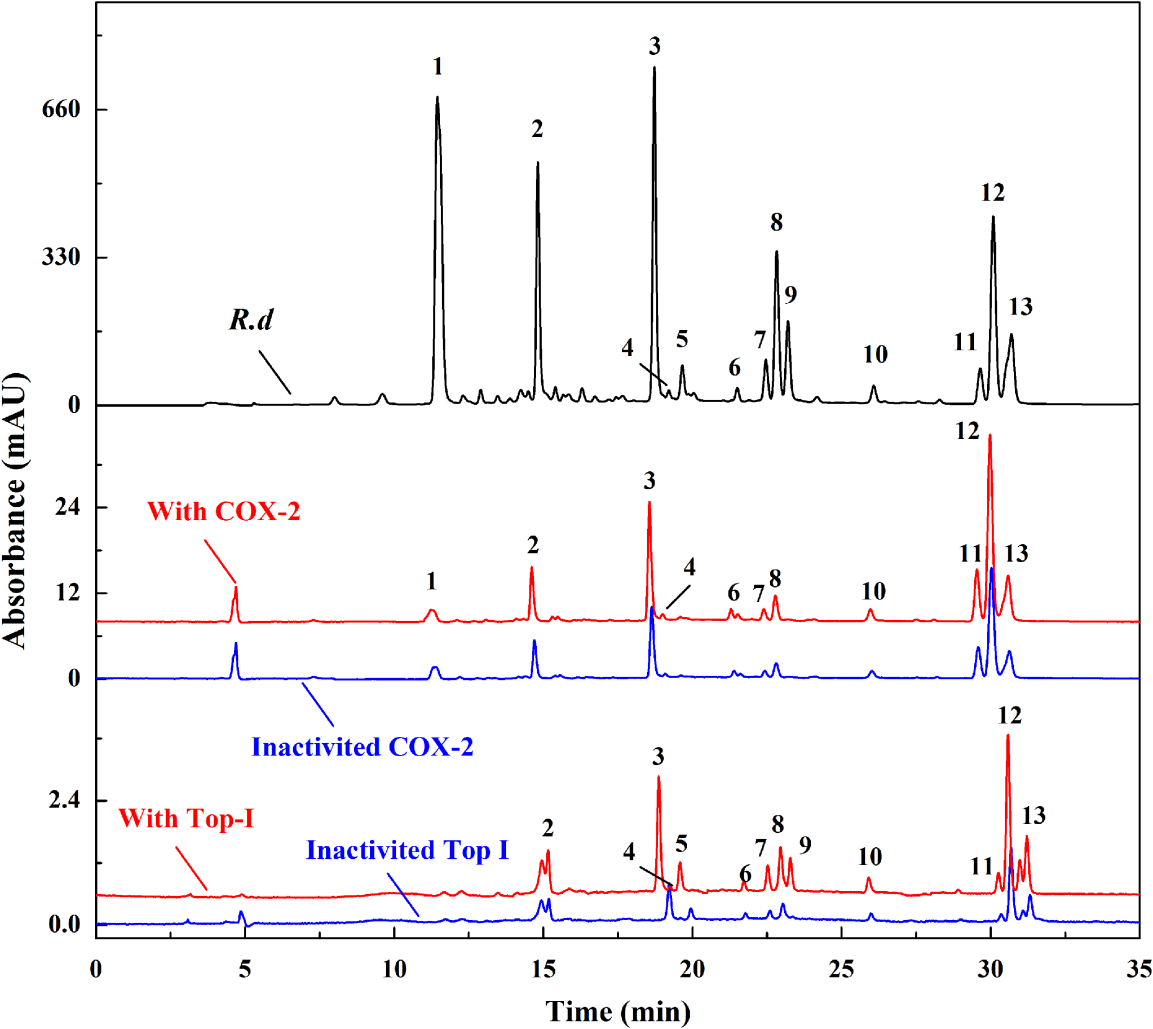
The HPLC profiles of the *R. davurica* extracts without ultrafiltration and with activated or inactivated COX-2 and Top I, respectively.

In this research area, our research group systematically screened out the potential anti-inflammatory components in the lotus plumule, a popular plant material with the same origin of medicine and food, by using the method of UF-LC/MS. For the first time, 12 flavonoids were quickly screened out as the potential cyclooxygenase 2 (COX-2, a key enzyme in the inflammatory reaction) inhibitors, in which structure-activity relationship indicated that the flavonoids *C*-glycosides showed comparable binding affinities to COX-2 compared to the flavonoids *O*-glycosides (Chen et al., 2019). In addition, topoisomerase I (Top I) and COX-2 were firstly employed as the dual-target bio-macromolecules to screen for the potential anti-tumor and anti-inflammatory components in the traditional medicinal plant *Rhamnus davurica* (*R. davurica*) with UF-LC/MS (Figure 3) (Chen et al., 2018b). As a result, 12 and 11 compounds in *R. davurica* extracts were screened out and identified as the potent Top I and COX-2 inhibitors, in which 10 compounds were indicated to be the common components responsible for the multi-component and multi-target way in *R. davurica*. In this study, UF-LC/MS technique displayed the characteristics of high specificity, rapidity, and simplicity, and the double-target screening of active components could reduce the false negative and false positive results of the selected components with high reliability.

Hydrogen-deuterium exchange mass spectrometry (HDX-MS) refers to the exchange of hydrogen atoms of the sample protein molecules with deuterated solvents (D_2_O, CD_3_OD, etc.), and the following detection of the mass shift of the enzymatic peptide by MS to determine the position of the protein sequence where the H/D atoms exchange occurs (Brock, 2012). Generally, hydrogen atoms that are prone to exchange between hydrogen and deuterium are located at the contact interface between protein and solution, and their exchange rate is faster than that of the hydrogen atoms that are located inside the protein structure or involved in hydrogen bond formation. Therefore, the structure and dynamic changes of proteins can be detected according to the different exchange rates of hydrogen and deuterium (Engen, 2009). When a small molecule inhibitor binds to a protein, it affects the hydrogen deuterium exchange rate of the hydrogen atom at the binding site, and thus the interaction region and intensity of the small molecule and the protein can be explored by the change of the hydroquinone exchange rate. As an effective tool for the study of protein structure and interaction kinetics, HDX-MS has been successfully used to analyze the interactions and mechanisms of action between proteins and small molecules (such as ATP and its analogs, small nucleic acids, small drug molecules, etc.) (Badireddy et al., 2011; Lorenzen and Pawson, 2014).

Zhang H. M. et al. (2010) used HDX-MS to study the interactions and mechanisms between drugs, imatinib and sunitinib, and the drug-resistant kinase receptor tyrosine kinase (KIT), and found that protein kinase mutant D816H changed KIT into an active form and thus could not bind with inhibitors. However, mutant V560D has two low-energy structures, one is loosely structured and similar to D816H, and the other is relatively compact and similar to wild-type KIT. Affected by these two low-energy structures, the inhibition effect of inhibitors on mutant V560D is between the wild-type and mutant D816H. Furthermore, Zhang J. M. et al. (2010) also succeeded in inspecting the binding positions, interactions and mechanisms of the allosteric inhibitor GNF-2 and tyrosine kinase Bcr-Abl with HDX-MS.

Ion mobility mass spectrometry (IM-MS) is a trace chemical analysis technology developed in the late 1960s and early 1970s by Cohen and Karasek (Armenta et al., 2011). The principle is based on the difference of the migration rate of different gas phase ions in the electric field to separate and characterize the chemical components, especially suitable for the trace detection of some volatile and semi-volatile organic compounds. This technique not only breaks through the limitations of the ion mobility spectrum, but also greatly expands the performance and application range of the mass spectrometer. Moreover, it can obtain the ion mobility and mass-to-charge ratio parameters of the samples and improve the accuracy of traditional mass spectrometry data; and at the same time as the specificity, the collisional cross-section (CCS) of the ions can be calculated, and the structural states of the samples can be obtained, which thus shows a great advantage in the identification of the unknown compounds (Pirok et al., 2017; Vautz et al., 2018).

Recently, a large number of literatures have reported the usages of the IM-MS for the analysis of the protein structures, protein high-throughput analysis, proteomics, protein-protein, and protein-small molecules interactions (Hines et al., 2017; Eyers et al., 2018). For instance, Benigni et al. (2016) established an IM-MS method for the detection of biological macromolecules and their complexes such as proteins, protein-nucleic acid complexes, and protein-protein complexes under denaturing and non-denaturing conditions, and for the first time the 150 kD protein and protein complexes were analyzed through the collision cross sectional area. Besides, Young et al. (2016) conducted the accurate and rapid analysis of the inhibitory effects of the protein aggregates and small molecule inhibition by virtue of the powerful analytical ability of the ESI-IM-MS, and identified two new small molecule inhibitors of Aβ 40 by taking the Aβ 40 amyloid as an example.

In addition, electrospray Fourier transform ion cyclotron resonance mass spectrometry (ESI-FT-ICR MS) can also be used to study the interactions of complex systems with proteins (Poulsen et al., 2006). Vu et al. (2008) investigated the interactions between 85 plant methanol extracts and carbonic anhydrase by ESI-FT-ICR MS, and screened out a specific binding inhibitor 6-(1s-hydroxy-3-methylbutyl)−7-methoxy-2h-chromen-2-one (a hydrolyzed product of coumarin).

### Applications of ESI-MS in the Interactions of Small Drug Molecules With Polypeptides

Peptides are also the targets for drug molecules in the body (Table 3). For example, toxic plaques produced by the aggregation of amyloid-β-peptides are one of the causes of Alzheimer's disease (AD), and drugs that show binding affinities to the amyloid-β-peptide segment (29-40) could prevent their aggregation and thus exert therapeutic effects (Skribanek et al., 2001). Bazoti et al. (2006, 2008) studied the interaction between Aβ polypeptide and oleuroprin using ESI-MS, and determined the binding site of oleuropein by enzymatic hydrolysis. The polypeptide side chain of the bacterial cell wall peptidoglycan is the binding site of some antibacterial drugs, which can block the synthesis of bacterial cell wall by binding to the end of the peptide chain, thus achieving the pharmacological effect of inhibiting the growth of bacteria. Lim et al. (1995) compared the relative acting forces of the two antibiotics, vancomycin, and ristocetin, on side chain polypeptides by ESI-MS, and found out that the binding strength of the former was higher than that of the latter. Additionally, some peptides can also be used as the models for proteins to study the interactions between drugs and proteins. For instance, Sarni-Manchado and Cheynier (2002) studied the binding properties of the catechin and their galacyl derivatives to the salivary proline-rich peptides by using ESI-MS, and also investigated the interactions between these components and gelatin proteins.

**Table 3:** Representative studies of ESI-MS technologies for the non-covalent interactions between small drug molecules and polypeptides.

**Subjects**	**Ligands**	**Type of MS**	**References**
Insulin	Phosphorylated daidzein derivatives	ESI-MS	Chen et al., 2008
Angiotensin peptide	Gold ion	FT-ICR-ESI-MS	Lee et al., 2012
18 α-amino acids	5 Ginsenosides	ESI-QT MS	Qu et al., 2009
Amyloid-β-peptide	Oleuropein	FT-ICR-ESI-MS	Bazoti et al., 2008
Amyloid-β-peptides	Nicotine, melatonin, 5- hydroxy-N-acetyltryptamine, daunomycin, doxorubicin	ESI-MS	Skribanek et al., 2001
Cell wall glycopeptides	Vancomycin, ristocetin	ESI-MS	Lim et al., 1995
Salivary proline-rich peptides	Catechin and its derived compounds	ESI-MS	Sarni-Manchado and Cheynier, 2002

### Applications of ESI-MS in the Interactions of Metal Ions With Proteins

Metal ions play an important role in the catalytic activities and structural stability of metalloenzymes and participate in many important biological processes (Carlton and Schug, 2011). *cis*-Platin, the second-generation platinum anticancer drug carboplatin, and the third-generation platinum anticancer drug oxaliplatin have been widely used in the treatment of malignant tumors such as testicular cancer, ovarian cancer, brain cancer, and bladder cancer (Le-Rademacher et al., 2017; Mukherjee et al., 2017). Therefore, to study the interactions between the metal anti-tumor drugs and proteins, and the effects of such interactions on cell intake, transport, metabolism and bioavailability of the drugs, is conducive to the structural design and optimization, the improvement of the anticancer activities and decrement the side effects of metal anticancer drugs, and some representative studies and findings in this field have been shown in the Table 4.

**Table 4:** Representative studies of ESI-MS technologies for the non-covalent interactions between small drug molecules and metal ions.

**Subjects**	**Ligands**	**Type of MS**	**References**
Ubiquitin	Platinum	NanoESI-Q-TOF MS	Hartinger et al., 2007
Ubiquitin	*cis*-Platin, *trans*-platin, oxaliplatin	ESI-FT-MS/MS	Hartinger et al., 2008b
Ubiquitin	*cis*-Platin	ESI-IM-MS	Williams et al., 2010
Chloroplast protein CP12	11 Metal ions	ESI-Qq-TOF MS	Delobel et al., 2005
Three beta-peptides	Zn^2+^	HPLC-ESI-TOF MS	Wortmann et al., 2005
HSA	Two organometallic ruthenium complexes	HPLC-ESI-Q-TOF MS	Hu et al., 2009
Transferrin, albumin, Ig G	*cis*-Platin	ESI-Q-TOF MS	Esteban-Fernández et al., 2008
Insulin	*cis*-Platin	ESI-IT/TOF MS	Li et al., 2016
Myoglobin	*cis*-Platin, *trans*-platin	ESI-Q-IT MS	Zhao and King, 2010

The HSA is the most abundant protein in human plasma, which binds to and transports a variety of endogenous metabolites and drugs. Currently, most of the platinum and ruthenium metal anticancer drugs are administered intravenously, and these drugs are widely bound to the HSA after entering the human body (Timerbaev et al., 2006; Hartinger et al., 2008a). These combinations are closely related to the drug activities, drug resistances and side effects, and the study of their interactions is of great significance for the structural design and optimization of this type of anticancer drugs. Hu et al. (2009) investigated the interactions between the HAS and two organometallic anticancer compounds [(η^6^-cymene) RuCl (en)] PF_6_, [(η^6^-biphenyl) RuCl (en)] PF_6_ (en = ethylenediamine) using HPLC-ESI-Q-TOF MS. In order to improve the coverage rate of peptide identification, the protein and protein-ruthenium complexes were denatured, reduced disulfide bonds, and blocked cysteine residues were treated prior to enzymatic hydrolysis of the protein sample to allow HSA trypsin to digest peptides. On this basis, it was found that [(η^6^-cymene) Ru (en)]^2+^ could form a coordination bond with the sulfhydryl group of the amino acid residue Cys34, and then induce the sulfhydryl oxidation to produce the sulfinic acid, which has an irreversible effect on the antioxidant function of HAS, while the [(η^6^-biphenyl) Ru (en)]^2+^ could not bind to the Cys34 due to the steric hindrance of the organic ligand biphenyl. In addition, the [(η^6^-cymene) RuCl (en)] PF_6_ and [(η^6^-biphenyl) RuCl (en)] PF_6_ could covalently bind to the residues of the His128, His247, His510 histidine, Met298 methionine on the HSA surface in the form of coordination bonds, and the former exhibited higher binding affinities than the latter. Therefore, this finding was of great significance for further optimizing the structure of ruthenium as an organometallic anticancer compound and improving the transport efficiency and bioavailability of drugs in the body.

Ubiquitin is a protein containing 76 amino acid residues and is widely found in the cytoplasm and nucleus. This protein plays a key role in the ubiquitination signaling pathway of cells, which is closely related to the proliferation of tumors and is a potential target of metal anticancer drugs in cancer cells (Hoeller et al., 2006; Williams et al., 2010). Hartinger et al. (2008b) studied the interactions among the *cis*-platin, oxaliplatin, and *tran*-splatin with ubiquitin protein using ESI-FT-MS/MS. After ionizing the platinum drugs-ubiquitin complexes, the protein complexes were directly cleaved by the collision-induced dissociation (CID) method. Analysis of platinum-containing fragments showed that the *cis*-platin and oxaliplatin were both bound to the ubiquitin Met1 residues, while the anti-platinum was bound to the peptide fragment of 19pro-ser-asp-thr-ile-glu24, but the bound amino acid residues could not be further determined. In addition, it was found that the platinum-containing protein fragments could be obtained by the CID and IRMPD (infrared multiphoton dissociation) pyrolysis methods, while could not be detected by the ECD (electron capture dissociation) pyrolysis methods. The reason may be that the platinum-containing fragments produced were neutralized due to the capture of electrons by platinum ions and could not be detected by mass spectrometry.

ESI source has been widely used in the field of protein-ligand non-covalent interactions, and many unstable biological macromolecule complex structures have been characterized. However, it requires the use of electric field and heating in the process of sample ionization. Besides, the removal of the solvent in the solution may cause the difference of the conformation of certain non-covalent protein-ligand complexes between the liquid state and gas phase of the mass spectrometer, and thus it is not suitable for some highly complicated protein-ligand complexes with thermal properties. In order to better maintain the natural conformation of proteins in solution, researchers have made some improvements to the ESI source, including electrosonic spray ionization (Wiseman et al., 2004) and cold spray mass spectrometry (CSI-MS) (Guo et al., 2009). By increasing the polarizabilities of the molecules by cooling the liquid spray and the solvent removal process, CSI-MS could promote the ionization of molecules and has been successfully applied to the study of supra-molecular systems that are heat unstable and cannot be analyzed by ESI-MS. Hence, the low-flow, ultra-high sensitivity nano-optimized ESI-MS, on the one hand, can achieve maximum sensitivity (up to f mol) with minimum sample consumption; on the other hand, the droplet volume produced by the spray is smaller, which speeds up the process of solvent removal, reduces the temperature and heat required for solvent removal, and facilitates the preservation of weak non-covalent interactions in the system (Yamaguchi, 2003).

### MALDI-MS and Its Applications in Ligand-Target Interactions

#### Characteristics of the MALDI-MS

**Figure 4:**
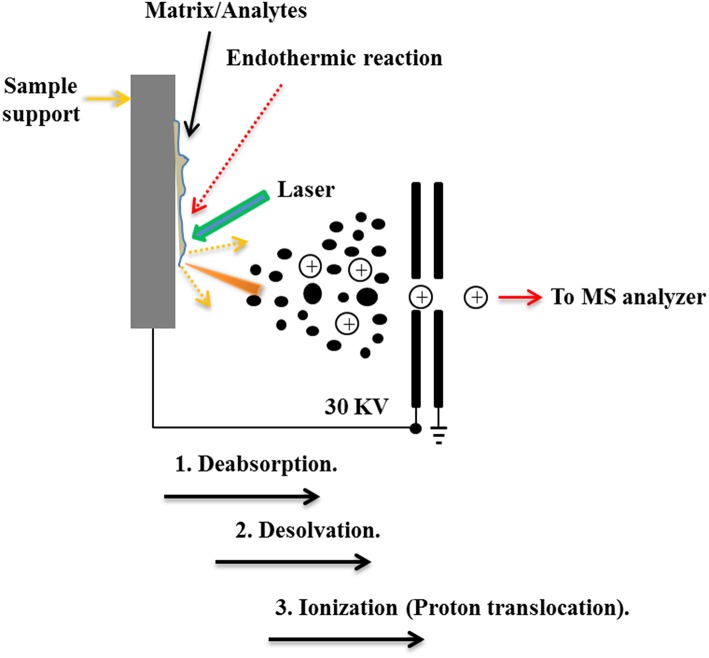
The schematic workflow of the typical MALDI ionization processes.

MALDI technology could make the non-volatile and thermal-unstable biological macromolecular compounds to form ions, which are detected at very low concentrations and used to characterize the spatial distributions and contents of the polypeptides and proteins in tissue sections (Mengistu et al., 2005). At this point, this technique could mix the small solid substrates with the analytes to be measured in a ratio of more than 5000:1. When irradiated with a laser, the matrix absorbs the laser energy and transmits it to the molecule to be tested, and which will be vaporized to form an ion. The matrix absorbs most of the laser energy and the fragment ions are reduced (Figure 4). Therefore, the main reason for the MALDI's breakthrough in macromolecular ionization is the use of substrates (Talian et al., 2007).

#### Applications of MALDI-MS in the Interactions of Small Drug Molecules With Proteins

Compared with ESI-MS, MALDI-MS is used much less in the studies of the interactions between the small drug molecules and the biological macromolecules, mainly because of the strong acidic matrix or organic co-solvents commonly used in sample preparation during MALDI-MS analysis, which is easy to cause the dissociation of non-covalent complexes. At the same time, the ionization process easily produces laser-induced polymers and matrix adducts, which often interferes with the detection of complexes (Bolbach, 2005). Although it is not easy for the MALDI-MS to directly detect complexes of the drugs and biological macromolecules, this analysis has a certain tolerance to salt solutions and buffers, which in a sense can better simulate the physiological environment. In addition, the MALDI-MS is fast, sensitive, simple and easy to automate, and thus has been prompted to study the interactions between the small drug molecules and the biological macromolecules with these advantages. Some representative studies and findings in this field have been shown in the following Table 5.

**Table 5:** Representative studies of MALDI-MS technologies for the non-covalent interactions between small drug molecules and biological macromolecules.

**Subjects**	**Ligands**	**Type of MS**	**References**
**DNA**			
Single-stranded DNA	Au nanoparticles	MALDI-TOF MS	Han et al., 2018
**PROTEINS**			
Avidin	Polymyxin B, angiotensin II and their biotinylated molecules	MALDI-TOF MS	Jorgensen et al., 2009
BSA	PGG	MALDI-TOF MS	Chen and Hagerman, 2004
Carboxypeptidase A	Three protease inhibitors (PCI, TCI, and LCI)	CL-MALDI-TOF MS	Yanes et al., 2006
**POLYPEPTIDES**			
Four tripeptides	2,5-dihyroxybenzoic acid, 3,5-dihyroxybenzoic acid, α-cyano-4-hydroxy-cinnamic acid	MALDI-MS	Ueno-Noto and Marynick, 2009
Angiotensin peptide	Gold ion	MALDI-TOF MS	Lee et al., 2012
Calmodulin	Melittin, substance P	IF-MALDI-MS	Wang et al., 2009
Aβ40 peptide	Promethazine	MALDI-MSI	McClure et al., 2013
**PROTEINS**			
HAS, Ig G, transferrin, BSA	Au nanoparticles	MALDI-TOF MS	Han et al., 2018
Insulin	*cis*-Platin	MALIDI-TOF/TOF MS	Li et al., 2016
Ubiquitin	Platinum	MALDI-MS	Hartinger et al., 2007
Cytochrome c oxidase	*cis*-Platin	MALDI-MSI	Aichler et al., 2013

Wang et al. (2009) introduced the intensity-attenuated MALDI-MS into the study of the interactions between the small molecules in the traditional Chinese medicine and proteins. To this end, the interactions between the alkaloids, calmodulin, and the melittin were studied by this method, and the relative binding strength of different drugs to targeted proteins was compared through titration and competitive experiments. Chen and Hagerman (2004) employed the MALDI-MS to directly detect the complexes formed by the bovine serum albumin (BSA) and tannin β-1, 2, 3, 4, 6-5-*O*-galacyl-D-glucose (PGG), and found that four PGG molecules could be bound to a single BSA molecule. Moreover, Yanes et al. (2006, 2007) studied the interactions between the ligand molecules and the biological macromolecules by the intensity-attenuated MALDI-MS. The basic principle of this method was to compare the changes of ionic strength of various ligand components (including negative control) in the samples before and after the addition of biological targeted molecules. Compared with the negative control, the ligand molecules were less selective after the addition of biological targeted molecules.

Mass spectrometry imaging (MSI) is a new type of molecular imaging technique that has received great attention in recent years. By scanning samples with ionized probes with different principles and structures, the *in-situ* desorption/ionization of the samples to be measured will be transmitted to the mass spectrum for detection, so as to obtain the mass spectrogram set associated with the spatial position of the samples (McDonnell et al., 2015). And, the tissue distribution image of each m/z is obtained by processing the mass spectrogram set obtained by mass spectrometry imaging software. Besides, the MALDI-MS forms the co-crystallization by mixing the tested components with the substrate capable of absorbing ultraviolet or infrared laser, and the substrate molecules absorb the laser energy and transfers part of it to the substance tested components, so as to achieve soft ionization of the tested components. By combining imaging processing software with mass spectrometry ion scanning technique, the MSI method could obtain the spatial distribution characteristics and content changes of the proteins and small molecules *in vivo*. It has the advantages of high chemical composition coverage and high throughput, and can realize imaging analysis of hundreds of compounds at a time (Spengler, 2015; Hansen and Lee, 2018). The MALDI is most widely used in the analysis of proteins and other macromolecules, but with the continuous development of MALDI technology, especially the rise of MALDI-MSI, it has been increasingly applied in the *in situ* analysis of lipids, small molecule metabolites, and small drug molecules (Bodzon-Kulakowska and Suder, 2016).

With the analysis of MALDI-MSI, McClure et al. (2013) found that after the injection of the antihistamine promethazine (25 mg/kg) in the tail vein, a large amount of Aβ40 peptide signals appeared in the brain tissue of transgenic mice with AD, and the ion signal strength of promethazine was three times that of wild mice, suggesting that Aβ40 peptide is an important biomarker for AD. In another study, Aichler et al. (2013) used MALDI-MSI to observe the protein expression differences in esophageal adenocarcinoma biopsy tissues before and after chemotherapy. The candidate proteins with obvious differences were identified by MS/MS for structure, and the results revealed that the chemo-sensitivity of tumor cells to *cis*-platin was significantly improved in the case of inhibiting the expression of cytochrome c oxidase. At present, the highest spatial resolution of MALDI-MSI could reach 1.4 μm (Kompauer et al., 2017).

Cross-linked mass spectrometry (CL-MS) is a rapidly evolving mass spectrometry technique for studying protein structures and interactions in recent years (Sinz, 2018). The cross-linking reaction involves covalently linking functional groups in one protein or between two proteins through the chemical crosslinking reagent. After the enzymatic hydrolysis, the peptides undergoing cross-linking reaction are enriched and isolated to determine the two adjacent cross-linking sites in space (Leitner, 2016). Generally, the cross-linking reagent is linked to two functional groups by a spacer arm of a specific length, which can be covalently reacted with the lysine, cysteine or other amino acids of the proteins depending on their properties (Lossl et al., 2016). In addition, the cross-linked peptides after enzymatic hydrolysis could be analyzed by the enzymatic hydrolysis of cross-linked proteins.

Based on the crystal structures of 1252 kinase-ligands, van Linden et al. (2014) obtained 85 amino acids with conserved sequences related to the ligand binding sites, and found that the amino acids such as glycine and lysine were distributed at multiple sites in the conserved region. The analysis of the lysine short-range microenvironment is conducive to the study of the interactions between the protein kinases, especially in the conserved regions, with small molecules. Moreover, the lysine in proteins is generally positively charged under the physiological conditions, and the interactions with adjacent amino acid residues, such as electrostatic interactions and hydrogen bonds, are one of the key factors to regulate protein structures and protein-protein interactions. Small molecule inhibitors often interact with the lysine residues of kinases to better target protein kinases and inhibit their activities, for example, inhibitors MK2206, PIA23, and K268 (Wu et al., 2010), K14 (Milburn et al., 2003) sites of AKT1 kinase, inhibitor imatinib and K271 site of ABL1 kinase (Nagar et al., 2002), inhibitor MP7 and K111 site of PDK1 kinase (Erlanson et al., 2011), and so on. Therefore, the analysis of lysine short-range microenvironment interactions plays an important role in studying the structural and functional regulation mechanisms of protein and protein complexes, and also has shown great potential in studying the interactions between the protein kinases and the small molecule inhibitors.

The main reason for MALDI's breakthrough in macromolecular ionization is the use of matrixes. Black et al. (2006) successfully analyzed polymers and peptides using a pencil lead as the matrix. Langley et al. (2007) also successfully applied this MALDI technique to other compounds. However, matrix effects in MALDI can lead to spatial matching errors (Chughtai and Heeren, 2010), and the sample needs to be introduced into a closed space with high vacuum during MS analysis, which limits the application of MALDI in the analysis of large samples such as whole animals. In 2004, Takáts et al. (2004) pioneered the breakthrough of DESI technique under the normal pressure conditions, which made the development of MS technique a promising step.

### Other Soft Ionization Technologies

#### Desorption Electrospray Ionization (DESI) and Its Applications in Ligand-Target Interactions

**Figure 5:**
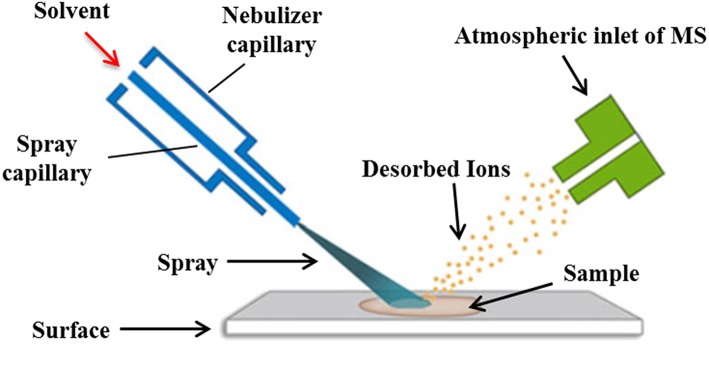
The schematic workflow of the typical DESI ionization processes.

DESI is a novel mass spectrometry ionization technique that combines the characteristics of ESI and desorption ionization (DI) techniques. Its ionization is achieved by spraying charged droplets generated by ESI onto the samples (Figure 5). Generally speaking, the sample is dissolved with appropriate solvent and then dropped onto the surface of insulating materials such as polytetrafluoroethylene (PTFE), polymethyl methacrylate (PMMA), etc., then the solvent is removed, and the sample is deposited on the surface of the carrier. The spray solvent used is first applied with a certain voltage and ejected from the inner casing of the atomizer. The high-speed N_2_ gas (linear velocity up to 350 m/s) ejected from the outer casing of the atomizer quickly atomizes and accelerates the solvent, causing the charged droplets to hit the sample surface. The sample is sputtered into the gas phase after being hit by high-speed droplets. At the same time, due to the scavenging and drying of N_2_ gas, the charged droplets containing sample are dissolvated and migrated along the ion transport tube at atmospheric pressure to the capillary at the front end of the mass spectrum, which is then detected by the detector of the mass spectrometer (Takáts et al., 2004; Zhu et al., 2018).

The sample ionization of DESI is carried out under normal pressure and open conditions. Since the sample does not need to be dissolved, the pretreatment is relatively simple, and the accuracy and *in situ* property of the sample are also ensured (Wiseman et al., 2008). At the same time, the DESI mass spectrum is more simplified and has unique advantages in the analysis of small molecules. Thereby, it has been widely used to study the interactions between small drug molecules and biological macromolecules (Table 6). In this field, Liu et al. (2013) employed the reactive liquid sample DESI to detect the non-covalent complexes of “ribonuclease A-cytidine nucleotide ligands” and “lysozyme-*N*-acetylglucosamine ligands,” and the results indicated that this “reactive” DESI technique could provide integrated information on the binding stiochiometry, selectivity, and kinetics of those two protein-ligand complexes. In another effort, the protein-carbohydrate interactions between lysozyme and L2, L3, and between a single chain variable fragment of Se155-4 and L4, L5, were studied by the liquid sample DESI-MS. It was found that the association constants of these protein-ligand complexes were in good agreement with values detected with the direct ESI-MS and isothermal titration calorimetry assays (Yao et al., 2015).

**Table 6:** Representative studies of DESI-MS and native MS technologies for the non-covalent interactions between small drug molecules and biological macromolecules.

**Subjects**	**Ligands**	**Type of MS**	**References**
Lysozyme	L2, L3	DESI-MS	Yao et al., 2015
Single chain variable fragment of Se155-4	L4, L5	DESI-MS	
Ribonuclease A	Cytidine nucleotide ligands	DESI-Q-TOF MS	Liu et al., 2013
Lysozyme	Acetyl chitose ligands	DESI-Q-TOF MS	
Lysozyme, trypsin, bovine β-lactoglobulin A, carbonic anhydrase II, concanavalin A, aptamer	Tri-*N*-acetylchitotriose, pefabloc, chlorothiazide, lauric acid, quinine	Native NanoESI-MS	Gavriilidou et al., 2015
Brentuximab vedotin	Brentuximab	Native MS, native IM-MS	Debaene et al., 2014
Adenosine, L-argininamide, cocaine binding aptamers	Sixteen ligands	Native ESI-MS	Gülbakan et al., 2018
Histidine-rich glycoprotein peptide 330	Zn^2+^	Native ESI-MS	Martin et al., 2018
Ubiquitin, lysozyme, myoglobin, RNase A	Cytidine 5′- diphosphate disodium salt, N,N',N”-triacetylchitotriose	NDX-MS	Zheng et al., 2018
Chicken egg white lysozyme, bovine pancreas trypsin, bovine β-lactoglobulin A, bCA II	Ammonium acetate, trimethylammonium acetate, triethylammonium acetate	Native ESI-MS	Zhuang et al., 2017

#### Native MS and Its Applications in Ligand-Target Interactions

Traditional protein analysis usually require denaturation of proteins by enzymatic hydrolysis, heating or adding high concentration of deformation agents (such as urea, guanidine hydrochloride, etc.), which will lead to the destruction of three-dimensional structure of proteins. In addition, for some commonly used separation methods, such as reversed-phase HPLC, the pH conditions of the mobile phase and the high concentration of organic compounds that are usually required also cause protein denaturation. When studying the non-covalent protein-ligand complexes, in order to prevent the non-covalent bonds from being destroyed by the above conditions, the native-denatured experimental conditions must be employed, thus producing the native MS technique.

The native MS combines certain soft ionization technique with MS, with the purpose to introduce the protein-ligand complexes into the gas phase environment of the mass spectrometer without destroying the non-covalent interactions, and to be recorded and analyzed completely. So there is no need to use complex protein engineering methods to analyze the protein-target system expressed at the physiological level, thereby completing the interactions of the non-covalent protein-ligand complexes and their structural and dynamic change process. In addition, for a more complex protein sample, the protein charge number will be reduced due to the decreased protons in the system when analyzed under the native-denatured conditions, thus the number of mass spectral peaks is reduced accordingly, and thereby causing the decreases of the overlap between different charge state interference, making it easier to obtain the molecular mass information for each component in the complex protein sample (Debaene et al., 2014). Some representative studies and findings in this field have been shown in the Table 6.

In general, ESI-MS is incompatible with non-volatile solution additives. Gavriilidou et al. (2015) studied the effects of ionic strength of ammonium acetate (AmAc) on the dissociation constants of six different non-covalent complexes using the native nano-MS, with a series of dilutions from 10 to 500 mM aqueous solutions, respectively. The results showed that the ionic strength exerted significant effect on the binding affinity of the non-covalent complexes. As the concentration of AmAc increased, the dissociation constant (K_d_) was affected by more than 50%. This work highlights the regulation of ions on non-covalent interactions and the importance of the selection of AmAc concentrations to quantify the receptor-target binding strengths. Besides, considering the inability of the native MS to discriminate the specific ligand-protein interactions from the non-specific interactions, Zheng et al. (2018) reported a native-denatured exchange MS (NDX-MS) method to recognize the changes from the native detection of non-covalent ligand-protein complexes to denatured analysis by employing three ligand-protein complexes of NAG3-lysozyme, CDP-ribonuclease and myoglobin. This NDX-MS method, remarkably with the distinguishing K_d_ dynamic profiles, could particularly recognize the specific ligand-protein interactions, and which could greatly aid to discovery specific protein targets for ligands of interest.

## Conclusion And Outlooks

In recent years, a number of reviews have been published on the applications of the ESI-MS and MALDI-MS in the studies of the non-covalent complexes. It can be seen that the ESI-MS dominates the studies of non-covalent complexes. Although the high sensitivity and relative molecular mass characterization range of the MALDI-MS are generally superior to the ESI-MS for the determination of biological macromolecules, its application in the detection of non-covalent complexes is limited by sample preparation conditions and high action energy. For example, the usage of the strong acidic matrix or organic co-solvents in the ionization process is generally detrimental to the formation of the non-covalent complexes. Moreover, the presence of laser-induced polymerization and matrix adduct formation often interferes with the detection of complexes, which makes MALDI-MS relatively less useful for the studies of the non-covalent complexes.

The non-covalent interactions can be classified as specificity and non-specificity, and the specific non-covalent complexes with biological functions are the focus of attention. The so-called complex peaks on the mass spectrum do not necessarily represent the actual complexes, and sometimes false positives occur. In order for the mass spectrometry data to truly reflect the non-covalent interactions of the system under test, it is necessary to use some experimental methods, such as the usage of the classical biochemical techniques of the enzymatic hydrolysis, chemical modification, etc., to change the sequences or properties of the biopolymers to weaken or destroy the non-covalent bonds, and then to compare the changes in the corresponding signals on the mass spectrum before and after the reaction to infer and verify the structures of the complexes. Secondly, different sample preparation techniques or different buffer systems need to be selected to optimize the test conditions in the experiment. It is also necessary to control the appropriate instrument conditions, especially the parameters of the ion source part, so as to reduce the dissociation of the non-covalent bonds and obtain the structural information of specific non-covalent complexes.

Proteins/enzymes play an important role in the life process, which are closely related to many malignant diseases such as tumors. Therefore, it is urgent to design and develop new small drug molecules targeting those proteins/enzymes. The aforementioned soft ionization MS-based methods could comprehensively, systematically and accurately study the protein/enzyme-small molecule interactions, and provide information such as the action sites (or regions) and the action intensity, so as to provide better help for drug design and disease treatment. With the development of soft ionization technologies, MS-based methods will continue to develop and improve, which will definitely promote the analysis and research of protein/enzyme-ligand interactions, so as to better serve for human health.

## Author Contributions

MG, JW, and BS conceived, designed, and supervised the manuscript. GC, MF, YL, ML, and NL collected the literatures, analyzed the data, and wrote the manuscript. All authors reviewed and approved the final manuscript.

### Conflict of Interest

The authors declare that the research was conducted in the absence of any commercial or financial relationships that could be construed as a potential conflict of interest.
